# Cross-Sectional Area Changes in the Total Cavopulmonary Connection Pathway and Its Impact on Liver Fibrosis

**DOI:** 10.3390/jcm15082930

**Published:** 2026-04-12

**Authors:** Nicole Piber, Christina Ruda, Thibault Schaeffer, Jonas Palm, Muneaki Matsubara, Stanimir Georgiev, Peter Ewert, Markus Krane, Jürgen Hörer, Masamichi Ono

**Affiliations:** 1 Department of Cardiovascular Surgery, TUM University Hospital, German Heart Center, 80636 Munich, Germany; 2 Department of Congenital and Pediatric Heart Surgery, TUM University Hospital, German Heart Center, 80636 Munich, Germany; 3 Division of Congenital and Pediatric Heart Surgery, University Hospital of Munich, Ludwig-Maximilians-Universität München, 81377 Munich, Germany; 4 Europäisches Kinderherzzentrum München, 80636 Munich, Germany; 5 Department of Congenital Heart Disease and Pediatric Cardiology, TUM University Hospital, German Heart Center, 80636 Munich, Germany; 6 DZHK (German Center for Cardiovascular Research), Partner Site Munich Heart Alliance, 806363 Munich, Germany

**Keywords:** total cavopulmonary connection, extracardiac conduit, Fontan-associated liver disease, liver fibrosis, Fibrosis-4 index

## Abstract

**Background/Objectives**: A known disadvantage of extracardiac Fontan is the absence of growth potential and potential late flow stagnation compared to lateral tunnel Fontan. This study investigates the differences in changes in the cross-sectional area and impact on liver fibrosis. **Methods**: The anteroposterior and lateral diameters of the Fontan pathways were measured using angiograms. Cross-sectional area and the indexed cross-sectional area were calculated, and their relation to Fibrosis-4 index was analyzed. **Results**: A total of 334 angiograms of 224 patients (212 extracardiac and 12 lateral tunnel Fontan) were evaluated. The median age at Fontan was 2.2 (Interquartile Range: 1.8–2.9) years. The median period from Fontan to angiogram was 3.3 (0.04–10.8) years. Cross-sectional areas remained unchanged in extracardiac Fontan patients and increased in lateral tunnel Fontan patients. The indexed cross-sectional areas in extracardiac Fontan patients decreased over time. The smallest indexed-cross-sectional areas were 200 mm^2^/m^2^ in extracardiac Fontan patients at 10 years postoperatively, whereas indexed cross-sectional areas in lateral tunnel Fontan patients were larger and more variable. Fibrosis-4 index increased time-dependently in both groups. Indexed cross-sectional area at the smallest level <156 mm^2^/m^2^ was identified as a risk factor for liver fibrosis. **Conclusions**: The Fontan pathway expanded in patients after lateral tunnel Fontan, whereas indexed cross-sectional area decreased over time after extracardiac Fontan. Importantly, in patients after extracardiac Fontan, narrowing of the Fontan pathway might be one of the risk factors for progression of liver fibrosis.

## 1. Introduction

The extracardiac conduit total cavopulmonary connection (EC-TCPC) is the method of choice for Fontan completion in patients with univentricular heart, rather than lateral tunnel (LT) TCPC, because EC-TCPC can be performed without cardiac arrest and has excellent perioperative outcomes even in high-risk cases [1,2,3,4,5,6,7,8]. Currently, EC-TCPC is typically performed on individuals aged 18 months and weighing 10 kg, using an 18mm Gore-Tex graft [5,6,7]. Despite numerous studies comparing these techniques, the differences in hemodynamic characteristics and outcomes remain controversial [9,10,11,12]. One of the main differences between LT-TCPC and EC-TCPC is the potential for growth of the TCPC pathway. The LT pathway is partially formed using the native atrial wall and has growth potential. The circumferential non-native EC conduit, however, cannot expand [13]. As TCPC patients reach adulthood, maintaining sufficient size of the TCPC pathway may be of critical importance for long-term survival, particularly as blood flow through the TCPC pathway increases with patient growth. Fontan-associated liver disease (FALD) is widely recognized as a potential complication after the Fontan procedure [13,14,15,16,17,18]. However, the association between the prevalence of fibrosis and vessel size after TCPC remains unclear.

The Fibrosis 4 (FIB-4) score is a blood-test-based, non-invasive method to assess liver scarring [19,20,21,22,23]. In patients with Fontan circulation, the FIB-4 score is used to assess liver fibrosis, occurring in Fontan-associated liver disease (FALD). It is associated with increased mortality and correlates with liver pressures and other markers of FALD [24,25,26].

This study aims to investigate the indexed cross-sectional area (i-CSA) of the TCPC pathway in patients after EC-TCPC and LT-TCPC, and to analyze the impact of i-CSA on liver fibrosis.

## 2. Materials and Methods

### 2.1. Patients and Data Collection

We conducted a retrospective chart review of all patients who underwent TCPC at the TUM University Hospital German Heart Center from January 1994 to December 2023. Postoperative cardiac catheterization data, angiographic data and further medical records were collected, including baseline morphology and demographics, as well as pre-, intra-, and post-procedural data, including weight, height, and body surface area (BSA) of the patients.

### 2.2. Surgical Techniques

LT-TCPC was performed between 1994 and 2002, according to the technique introduced by de Leval et al. and a polytetrafluoroethylene patch (Gore-Tex, WL Gore&Associates, Newark, DE, USA) was used for the intra-atrial baffle [27]. In January 1999, EC-TCPC was introduced using a non-ringed polytetrafluoroethylene graft (Gore-Tex, WL Gore&Associates, Newark, DE, USA) and became our standard procedure since May 2002 [7]. Cardioplegic arrest was only used for patients who required intracardiac procedures. Fenestration was not routinely performed, only in high-risk patients, such as those with single-lung Fontan or reduced ventricular function [7].

### 2.3. Measurement of CSA in the TCPC Pathway

**Figure 1:**
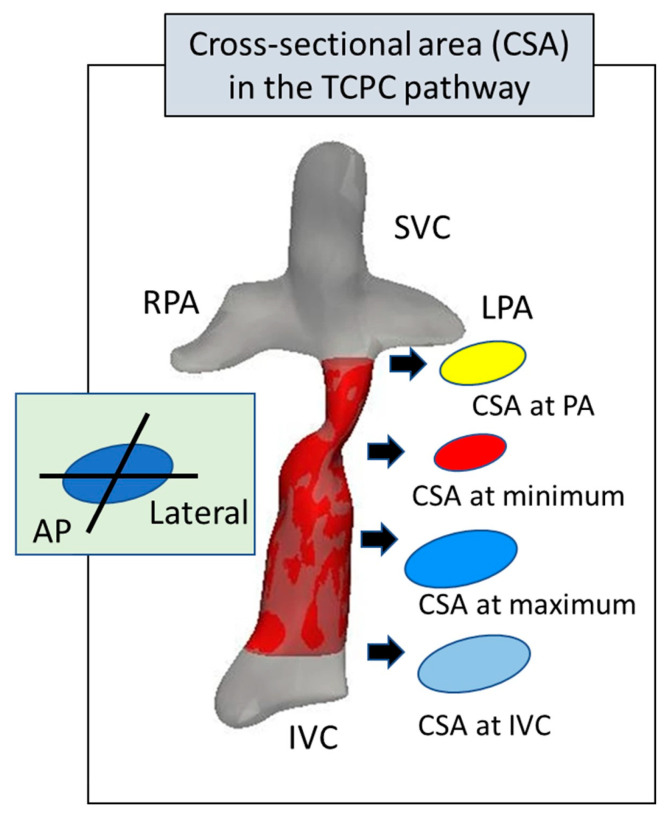
A figure demonstrating the measurement of CSA. TCPC, total cavopulmonary connection; PA, pulmonary artery; RPA, right pulmonary artery; SVC, superior vena cava; LPA, left pulmonary artery; CSA, cross-sectional area; AP, anterior–posterior.

Cardiac catheterization was not routinely performed after TCPC; however, it was performed when indicated. Using the TCPC pathway angiogram from the injection at the inferior vena cava (IVC), the diameters of the TCPC pathways were measured in four levels: the largest level, the smallest level, the PA anastomosis level, and the IVC anastomosis level. At each level, the diameters were measured in two ways: anteroposterior (AP) and lateral (Figure 1). The CSA in the TCPC pathway was calculated using the following formula:

CSA = π (AP diameter + lateral diameter)2/4)

CSA was calculated at all four levels in the TCPC pathway. The indexed CSA (i-CSA), defined as CSA divided by body surface area (BSA), was also determined in each patient.

### 2.4. Fibrosis-4 Index

FIB-4 index for liver fibrosis was calculated using the following formula:

FIB-4 = Age (years) × AST (U/L)/[Platelet Count (10^9^/L) × √(ALT (U/L))].

### 2.5. Impact of i-CSA on Liver Fibrosis

The impact of i-CSA on liver fibrosis was evaluated. The i-CSA was used as a continuous variable and dichotomized (less than 25 IQR). The FIB-4 score was used as a marker for liver fibrosis, and FIB-4 > 0.17 was defined as the cutoff value, as it marks 25% of the interquartile range (IQR).

### 2.6. Statistical Analysis

Categorical variables are presented as absolute numbers and percentages. A chi-squared test is used for categorical data. Continuous variables are expressed as medians with interquartile ranges (IQRs). An independent sample Student’s *t*-test is used to compare normally distributed variables. The Mann–Whitney U test is used for variables that were not normally distributed. A logistic regression model is used to evaluate whether i-CSA is a risk for liver fibrosis. Results are shown with odds ratio (OR) and 95% confidence interval (CI). Significant association with the outcome is defined as *p* < 0.05. Data analysis is performed using SPSS version 28.0 for Windows (IBM, Ehningen, Germany) and R statistical software version 4.2.3 (R Foundation for Statistical Computing, Vienna, Austria).

## 3. Results

### 3.1. Patient Characteristics

A total of 650 patients underwent TCPC at our institution between January 1994 and December 2023. Among them, 224 patients (212 EC-TCPC patients and 12 LT-TCPC patients) underwent at least one cardiac catheterization and angiogram, resulting in a total of 334 angiograms being evaluated. Characteristics of all 224 patients included in this study are shown in Table 1. The median age and weight at TCPC were 2.2 (1.8–2.9) years and 11.4 (10.5–13.0) kg. Hypoplastic left heart syndrome (HLHS) was the most frequent primary diagnosis (n = 84, 37.3%), only being diagnosed in the EC-TCPC group (*p* = 0.006), followed by univentricular heart in 43 patients (UVH), occurring more often in the LT-TCPC group (41.7% vs. 17.9%, *p* = 0.042). A dominant right ventricle was observed in 143 (63.8%), commonly in the EC-TCPC group (65.5% vs. 33.3%, *p* = 0.024). Transposition of the great arteries (TGAs) was diagnosed in a total of 65 patients, mainly in the LT-TCPC group (26.4% vs. 75.0%, *p* < 0.001). Within the EC-TCPC and LT-TCPC groups, the proportion of patients with HLHS (39.6% vs. 0%, *p* = 0.006) and previous Norwood procedure (61.8% vs. 16.7%, *p* = 0.002) was higher in the EC-TCPC group. However, pulmonary artery banding (PAB), as a form of stage I palliation, also showed a statistically significant association, with a higher rate in the LT-TCPC group (33.3% vs. 11.3%, *p* = 0.025). Stage II palliation was performed in 210 patients (93.8%) and was also associated more often with the EC-TCPC group (96.7% vs. 41.7%, *p* < 0.001). Age at the time of bidirectional cavopulmonary shunt (BCPS) was higher in the LT-TCPC than in the EC-TCPC (*p* = 0.009). In 212 patients with EC-TCPC, the conduit size ranged from 14 mm (n = 1) to 22 mm (n = 4). The most common conduit size was 18 mm, which was used in 189 patients.

### 3.2. Early Outcomes

The median length of intensive care unit (ICU) stay and hospital stay were 7 (5–11) days and 22 (17–33) days, respectively. There was no difference in the length of the ICU stay (median 9 vs. 8 days, *p* = 0.681) and the hospital stay (median 27 vs. 33 days, *p* = 0.207) between the EC-TCPC and LT-TCPC groups. There was no difference in the incidence of prolonged pleural effusion (62.6 vs. 50.0%, *p* = 0.384), chylothorax (30.3 vs. 16.7%, *p* = 0.313), or ascites (23.7 vs. 16.7%, *p* = 0.575).

### 3.3. CSA/i-CSA of TCPC Pathway

The values of CSA and i-CSA, calculated using a total of 334 angiograms, are shown in Table 2. The median period from TCPC to angiogram was 3.3 (IQR: 0.04–10.8) years. The median CSA was 238 mm^2^ at PA anastomosis site, 185 mm^2^ at the narrowest site, 234 mm^2^ at the largest CSA, and 180 mm^2^ at IVC anastomosis. The median i-CSA was 292 mm^2^/m^2^ at PA anastomosis site, 235 mm^2^/m^2^ at the narrowest site, 313 mm^2^/m^2^ at the largest CSA, and 212 mm^2^/m^2^ at IVC anastomosis site. When the variables are compared in patients with EC-TCPC and LT-TCPC, CSA and i-CSA were larger in patients with LT-TCPC than in patients with EC-TCPC at each level.

**Figure 2:**
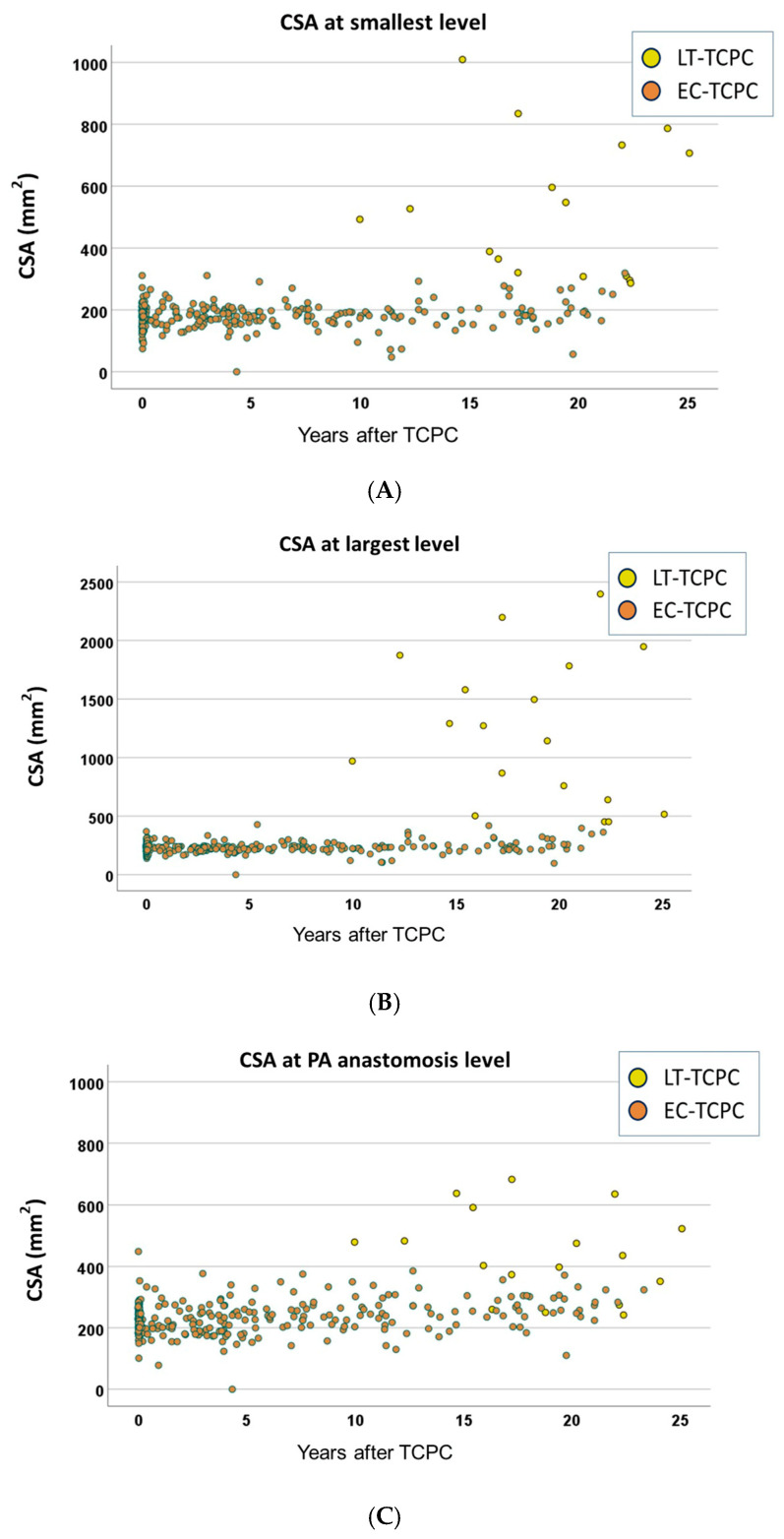

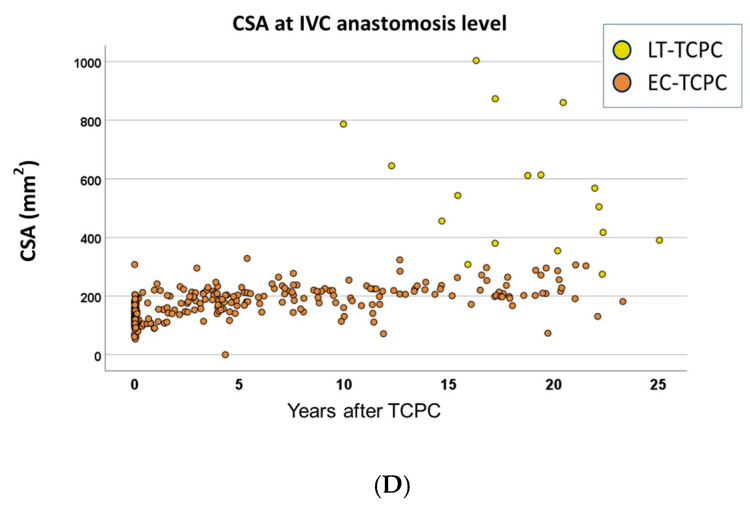
The relation of CSA and time following TCPC. (**A**): the smallest level, (**B**): the largest level, (**C**): PA anastomosis level, (**D**): IVC anastomosis level. CSA, cross-sectional area; TCPC, total cavopulmonary connection; PA, pulmonary artery; IVC, inferior vena cava.

**Figure 3:**
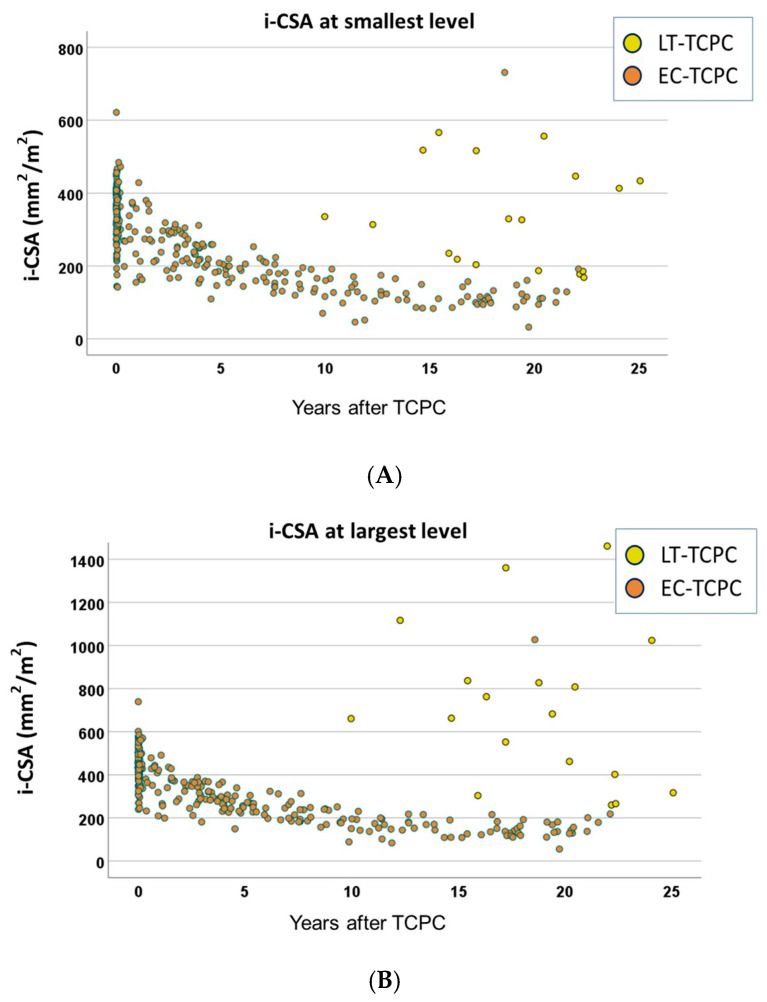

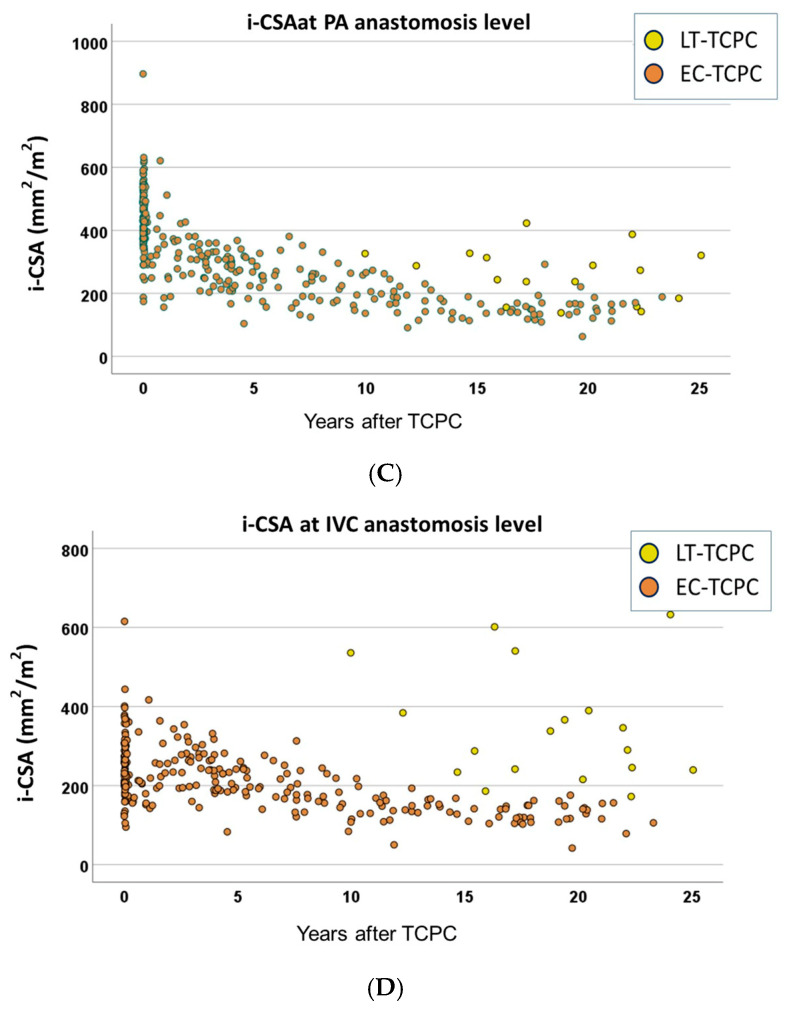
The relation of i-CSA and time following TCPC. (**A**): the smallest level, (**B**): the largest level, (**C**): PA anastomosis level, (**D**): IVC anastomosis level. CSA, cross-sectional area; TCPC, total cavopulmonary connection; PA, pulmonary artery; IVC, inferior vena cava.

The relations of the period from TCPC to angiogram and CSA are shown in Figure 2. The CSAs in EC-TCPC patients remained unchanged, as expected, whereas those in LT-TCPC patients were larger and more variable. The relations of the period from TCPC to angiogram and i-CSAs are shown in Figure 3. The i-CSAs in EC-TCPC patients decreased over time. The smallest i-CSAs were 200 mm^2^/m^2^ or less in most of the EC-TCPC patients at 10 years postoperatively (Figure 3A), whereas i-CSAs in LT-TCPC patients were larger and more variable than in EC-TCPC patients.

### 3.4. FIB-4 Score

**Figure 4:**
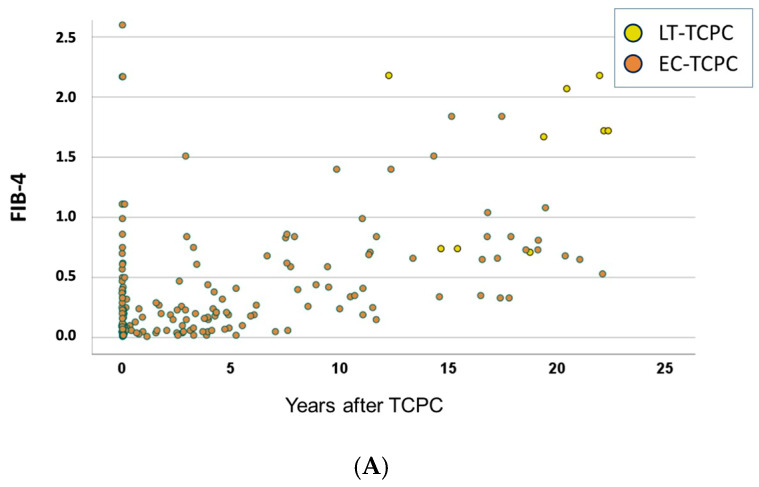

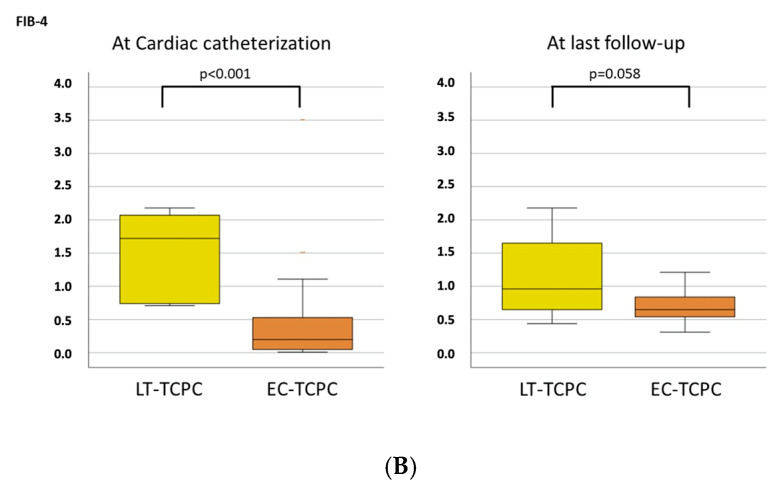
Fib-4 values regarding the time after TCPC and types of TCPC. (**A**) The relation of Fib-4 and time following TCPC. (**B**): Comparison of Fib-4 between EC-TCPC and LT-TCPC.

The correlation of the period from TCPC to angiogram and FIB-4 is shown in Figure 4A. FIB-4 levels increased time-dependently in both EC-TCPC and LT-TCPC patients. The FIB-4 values of LT-TCPC and EC-TCPC are shown in Figure 4B. When FIB-4 was compared at the time of cardiac catheterization, FIB-4 in LT-TCPC patients was significantly higher than that in EC-TCPC (*p* < 0.001). When FIB-4 was compared at the last follow-up, there was no significant difference between the groups (*p* = 0.058).

### 3.5. Impact of i-CSA on Liver Fibrosis

**Figure 5:**
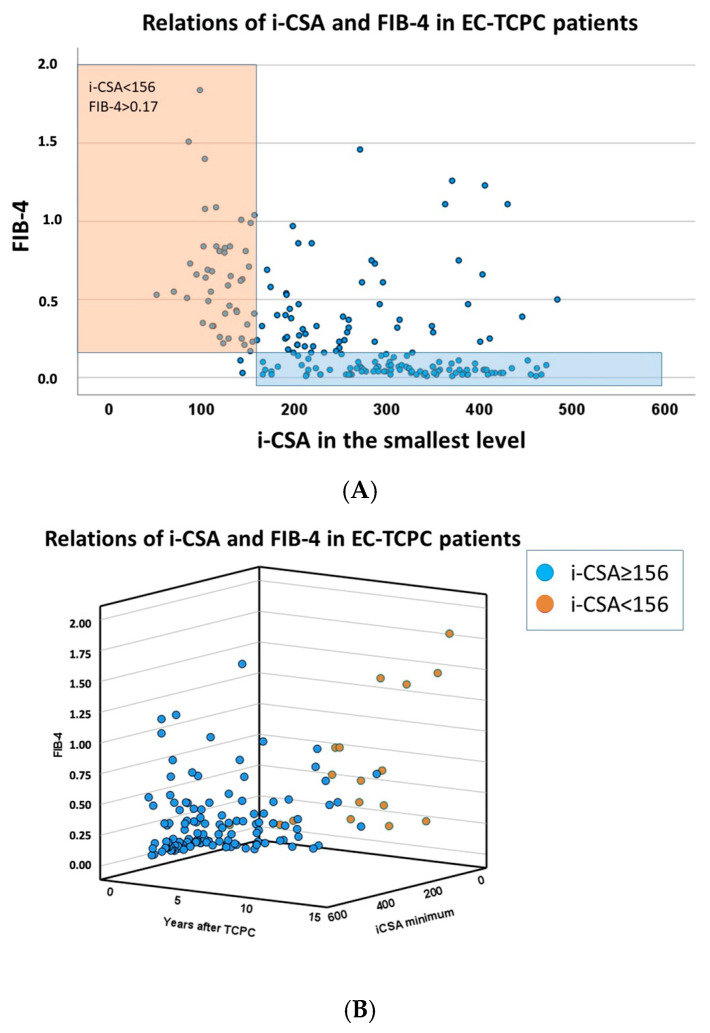
The relationship between Fib-4 and i-CSA. (**A**) Two-dimensional graphic demonstrating the relationship between Fib-4 and i-CSA. Red background indicating i-CSA < 156 and FIB-4 > 0.17, blue background indicating i-CSA > 156 and FIB-4 < 0.17 (**B**) Three-dimensional graphic demonstrating the relationship between Fib-4 and i-CSA. CSA, indexed cross-sectional area; FIB-4, fibrosis-4; EC-TCPC, extracardiac total cavopulmonary connection.

The influence of small i-CSAs on the elevation of FIB-4 was evaluated in 212 EC-TCPC patients (Table 3). FIB-4 value of 0.17 (25 IQR) was used as the cutoff value. Small i-CSAs at all four levels were risks for elevated FIB-4 as continuous variables. As for the dichotomized variables, i-CSA at PA anastomosis <193 mm^2^/m^2^ (OR 14.1, *p* < 0.001), i-CSA at smallest level <156 mm^2^/m^2^ (OR: 35.6, *p* < 0.001), and i-CSA at IVC anastomosis <157 mm^2^/m^2^ (OR: 7.0, *p* < 0.001) were identified as risk factors by univariate model. Multivariable model revealed i-CSA at the smallest level being <156 mm^2^/m^2^ as an independent risk factor for liver fibrosis. The 2-dimensional relation of i-CSA at the smallest level and FIB-4 is shown in Figure 5A, and the 3-dimensional relation of time after TCPC, i-CSA at the smallest level, and FIB-4 is shown in Figure 5B.

## 4. Discussion

### 4.1. Narrowing of the TCPC Pathway

The size of EC-TCPC vessel does not align with somatic growth [28,29]. This study was able to objectify this relation by measuring the i-CSA. As a result of a narrow conduit, pulmonary artery growth fails to match the increase in body surface area after Fontan operation, and pulmonary artery size is associated with functional clinical status in Fontan circulation [30,31,32]. EC-TCPC conduits in this study were significantly smaller than LT-TCPC. Due to sustained flow efficiency in LT-TCPC compared to EC-TCPC, a lower rate of Fontan failure was detected in LT-TCPC in our study [33]. EC-TCPC pathways tend to become more stenotic over time, whereas the LT-TCPC tends to dilate [34]. In this study, the decrease in i-CSA of EC-TCPC continued until the latest follow-up, with concerningly small i-CSA after 10 years. At the same time, the LT-TCPC showed a gradual expansion, which is consistent with previous studies [28,33,34].

### 4.2. TCPC Conduit i-CSA and Liver Fibrosis

The i-CSA was larger in patients with LT-TCPC at each measured level and expanded over time, whereas in EC-TCPC the i-CSA decreased over time. Although our research suggested that the LT-TCPC patients tend to have higher FIB-4 values than the EC-TCPC patients, this finding may be biased due to the higher average age of the LT group, as age is a component of the FIB-4 score and directly influences its value. When examining EC-TCPC patients alone, we noted that an i-CSA smaller than 156 mm^2^/m^2^ has a higher risk of having an FIB-4 value over 0.17. Previously, various methods were employed, including liver ultrasound, laboratory parameters and liver biopsy, to diagnose liver fibrosis [24,25,26]. Rijnberg et al. raised the concern that the conduit sizes used nowadays, such as 16–20 mm for the EC-TCPC, might be too small for older Fontan patients [35]. An undersized conduit leads to elevated resistance and, therefore, to a higher central venous pressure, resulting in structural and functional remodeling of the liver parenchyma, a key mechanism in FALD [36]. High central venous pressure is dependent on the geometry, including size and morphology rather than material of the vessel [37]. Fib-4 values over 0.17 can be seen as a clinical precursor for narrowing of the i-CSA and imminent liver fibrosis. Based on these observations, alternative surgical strategies should be explored, such as implanting other, non-rigid materials, including dilatable grafts or tissue-engineered grafts with growth potential [38].

### 4.3. Limitations

The retrospective single-center study design has the disadvantage of inherent bias. As the number of patients, especially LT-TCPC patients, is limited, the link between liver fibrosis and conduit size is weak. LT-TCPC was discontinued at our institution in 2002. The FIB-4 score is an established marker for liver fibrosis, but lacks diagnostic accuracy compared to liver biopsy or additional assessments. Secondly, the FIB-4 score is age-dependent, which may be a disadvantage in this study as age varies between the two groups.

## 5. Conclusions

This study examines vessel size in Fontan patients in correlation to liver fibrosis by analyzing serial CMR data. The TCPC pathway enlarged in patients after LT-TCPC, whereas the indexed conduit size decreased over time in those after EC-TCPC. Importantly, FIB-4 values were higher in patients after LT-TCPC, compared to those after EC-TCPC. In patients after EC-TCPC, narrowing of the TCPC pathway is found to be one of the risk factors for the progression of liver fibrosis. While growth in absolute terms was observed in all LT-TCPC vessels, normalized diameters generally decreased, indicating that although vessel growth did occur, it was not proportionate to somatic growth.

## Figures and Tables

**Table 1:** Patient’s characteristics.

Variables		Total Cases	EC-TCPC	LT-TCPC	*p*-Value
Number of patients		224	212 (94.6)	12 (5.4)	
Age at TCPC (years)		2.2 (1.8–2.9)	2.2 (1.8–2.8)	4.4 (2.4–7.8)	0.135
Weight at TCPC (kg)		11.4 (10.5–13.0)	11.4 (10.6–13.0)	15.9 (10.9–21.7)	0.119
Fenestration		21 (9.4)	13 (6.1)	8 (66.7)	<0.001
Primary Diagnosis					
	HLHS	84 (37.3)	84 (39.6)	0 (0.0)	**0.006**
	UVH	43 (19.2)	38 (17.9)	5 (41.7)	**0.042**
	DILV	28 (12.4)	26 (12.3)	2 (16.7)	0.654
	TA	24 (10.7)	22 (10.4)	2 (16.7)	0.493
	UAVSD	13 (5.8)	13 (6.1)	0 (0.0)	0.377
	PAIVS	7 (3.1)	7 (3.3)	0 (0.0)	0.522
	ccTGA	9 (4.0)	9 (4.2)	0 (0.0)	0.466
	Others	17 (7.6)	14 (6.6)	3 (25.0)	**0.019**
Dominant right ventricle (RV)		143 (63.8)	139 (65.6)	4 (33.3)	**0.024**
Associated cardiac anomaly					
	TGA	65 (29.0)	56 (26.4)	9 (75.0)	**<0.001**
	DORV	24 (10.7)	21 (9.9)	3 (25.0)	0.100
	CoA	40 (17.9)	37 (17.5)	3 (25.0)	0.507
	Dextrocardia/Situs Inversus	15 (6.7)	15 (7.1)	0 (0.0)	0.340
	Heterotaxy	20 (8.9)	20 (9.4)	0 (0.0)	0.265
	TAPVC/PAPVC	19 (8.5)	19 (9.0)	0 (0.0)	0.278
	Systemic venous return anomaly	24 (10.7)	24 (11.3)	0 (0.0)	0.217
Stage I palliation					
	Norwood/DKS	133 (59.4)	131 (61.8)	2 (16.7)	**0.002**
	AP Shunt	48 (21.4)	43 (20.3)	5 (41.7)	0.079
	PAB	28 (12.5)	24 (11.3)	4 (33.3)	**0.025**
	PDA stent	16 (7.1)	16 (7.5)	0 (0.0)	0.323
Stage II palliation		210 (93.8)	205 (96.7)	5 (41.7)	**<0.001**
	Age at BCPS (months)	4.7 (3.5–6.9)	4.6 (3.5–6.7)	18.3 (8.3–40.9)	**0.009**

Data are presented by N(%) or median (IQR). BCPS, bidirectional cavopulmonary shunt; TCPC, total cavopulmonary connection; HLHS, hypoplastic left heart syndrome; UVH, univentricular heart; TA, tricuspid atresia; DILV, double inlet left ventricle; ccTGA, congenitally corrected TGA; PAIVS, pulmonary atresia and intact ventricular septum; UAVSD, unbalanced atrioventricular septal defect; TGA, transposition of the great arteries; DORV, double outlet right ventricle; CoA, coarctation of the aorta; TAPVC, total anomalous pulmonary venous connection; PAPVC, partial anomalous pulmonary venous connection; AP, aorto-pulmonary; PAB, pulmonary artery banding; PDA, patent ductus arteriosus.

**Table 2:** Cross-sectional area (CSA) in the TCPC pathway.

Variables		Total Cases	EC-TCPC	LT-TCPC	*p*-Value
Number of patients		224	212 (94.6)	12 (5.4)	
Number of angiograms		334	316 (94.6)	18 (5.4)	
**Variables at angiogram**					
	Period from TCPC (y)	3.3 (0.04–10.8)	2.9 (0.04–9.1)	19.1 (15.8–22.2)	<0.001
	Weight (kg)	19.4 (12.1–42.1)	18.1 (12.0–37.1)	60.5 (56.2–72.9)	0.038
	Height (cm)	110 (89–155)	108 (88–144)	169 (163–174)	<0.001
BSA (m^2^)		0.76 (0.54–1.37)	0.72 (0.53–1.20)	1.67 (1.63–1.83)	<0.001
**CSA (mm^2^)**					
	PA anastomosis	238 (201–271)	234 (200–265)	436 (313–557)	<0.001
	Minimal CSA	185 (163–206)	183 (161–202)	537 (318–799)	<0.001
	Maximal CSA	234 (214–259)	232 (213–252)	1208 (609–1806)	<0.001
	IVC anastomosis	180 (133–216)	177 (128–210)	556 (388–805)	<0.001
**i-CSA (mm^2^/m^2^)**					
	PA anastomosis	292 (191–399)	297 (193–405)	273 (171–324)	0.026
	Minimal CSA	235 (160–334)	233 (156–326)	328 (200–464)	<0.001
	Maximal CSA	313 (211–440)	304 (199–429)	673 (381–884)	<0.001
	IVC anastomosis	212 (160–266)	207 (263–157)	314 (238–426)	0.001

Values are expressed in N (%). TCPC, total cavopulmonary connection; EC-TCPC, extracardiac total cavopulmonary connection; LT-TCPC, lateral tunnel total cavopulmonary connection; BSA, body surface area; CSA, cross-sectional area; PA, pulmonary artery; IVC, inferior vena cava; i-CSA, indexed cross-section area.

**Table 3:** Impact of i-CSA on liver fibrosis.

Variables		Univariable			Multivariable	
	OR	95% CI	*p*-Value	OR	95% CI	*p*-Value
Continuous variables						
i-CSA at PA	0.993	0.991–0.996	<0.001			
i-CSA min	0.990	0.987–0.993	<0.001			
i-CSA max	0.992	0.989–0.994	<0.001	0.988	0.985–0.992	<0.001
i-CSA at IVC	0.996	0.992–1.000	0.034			
Dichotomized (<25 IQR)						
i-CSA at PA < 193	14.1	4.8–41.3	<0.001			
i-CSA min < 156	35.6	8.3–151.9	<0.001	33.6	7.9–143.5	<0.001
i-CSA max < 199	-	-	0.997			
i-CSA at IVC < 157	7.0	2.9–16.7	<0.001			

i-CSA: indexed cross-section area; PA: pulmonary artery; IVC: inferior vena cava; IQR: interquartile range; OR: odds ratio; CI: confidence interval.

## Data Availability

The data are available from the corresponding author upon reasonable request.

## Abbreviations

The following abbreviations are used in this manuscript:

CI	confidence interval
CSA	cross-sectional area
EC	Extracardiac
FALD	Fontan-associated liver disease
FIB-4	Fibrosis-4
i-CSA	indexed cross-sectional area
ICU	intensive care unit
IQR	interquartile range
LT	lateral tunnel
OR	odds ratio
PA	pulmonary artery
TCPC	total cavopulmonary connection
BCPS	bidirectional cavopulmonary shunt
